# Updating, Fast and Slow: Items, but Not Item-Context Bindings, are Quickly Updated Into Working Memory as Part of Response Selection

**DOI:** 10.5334/joc.257

**Published:** 2023-01-17

**Authors:** Yoav Kessler, Nitzan Zilberman, Shalva Kvitelashvili

**Affiliations:** 1 Department of Psychology and School of Brain Sciences and Cognition, Ben-Gurion University of the Negev, Beer-Shava, 8410501, Israel; 2 Department of Psychology and Zlotowski Center for Neuroscience, Ben-Gurion University of the Negev, Beer-Sheva, 8410501, Israel

**Keywords:** Working memory, Executive functions, Attention

## Abstract

It is commonly held that attending to items facilitates their encoding into working memory (WM). This implies that the content of WM is updated with new input as a consequence of directing attention to it. On the other hand, abundant research shows that WM updating is rather slow and effortful, suggesting that shielding WM representation against incoming input, rather than its updating, is the default. To resolve this discrepancy, we suggest that while updating item-to-context associations is costly, updating a single item is fast and is automatically carried out as part of directing attention to items, for example as part of response selection. Participants performed a choice-RT task, in which stimuli appeared within frames, and needed to update their WM with the most recent red item that appeared in each frame. The need for updating was manipulated, so that some trials required updating and others did not. Experiment 1 (N = 25) showed that updating was slower than not updating with a set-size of two items, that required item-context binding, but faster when the set-size only involved one item. Experiment 2 (N = 28) replicated this finding. Experiment 3 (N = 20) showed that the slower no-update RTs are due to the removal of erroneously updated information. In contrast to previous findings, these results suggest that updating can be effortless and obligatory.

To keep track of our ever-changing environment, we constantly need to update our working memory (WM) with goal-relevant information. WM updating is considered a core executive function (Miyake et al., 2000), which plays an essential part in goal-directed behavior. Over the past three decades, a large body of research has investigated the behavioral and neural underpinning of WM updating. A key finding is that updating is generally controlled, slow and costly. Studies that manipulated the need for updating observed longer response times (RTs) in trials that required updating was longer than in no-update trials (Kessler & Meiran, 2006, 2008; Kessler & Oberauer, 2014, 2015). An influential modeling framework, the pre-frontal basal-ganglia WM model (PBWM), holds that WM is shielded from the environment by a gate, that enables robust maintenance within WM by blocking incoming input. The gate is closed by default and can be transiently opened when required to enable WM updating (Frank et al., 2001). Indeed, experimental paradigms that enable a finer-detailed analysis of WM updating latency revealed that this cost is due to the operation of several distinctive and time-consuming sub-process involved, including opening the gate to WM (Rac-Lubashevsky & Kessler, 2016a, b), substitution (Ecker, Lewandowsky, Oberauer & Chee, 2010), and removing outdated information (Ecker, Lewandowsky & Oberauer, 2014).

However, the latency of WM updating, its proneness to error (Frischkorn, von Bastian, Souza & Oberauer, 2022), and its associated self-reports of mental effort (Westbrook, Kester & Braver, 2013) are all at odds with the common view that attention plays a role in determining the contents of WM. According to this view, orienting attention toward stimuli lead to their automatic encoding into WM (Cowan, 1999). While mere attention is not a sufficient condition for encoding items into WM (Chen & Wyble, 2015), directing attention to items facilitates this process (Oberauer, 2019). This view implies that updating may take place as part of, or as a result of, other attention-consuming processes, such as response selection (Oberauer, Souza, Druey & Gade, 2013; Szmalec, Vandierendonck & Kemps, 2005). It follows that updating is often the *default* outcome of attending to items and hence should be relatively effortless and automatic, rather than slow and effortful. If so, then it is the *resistance* to updating, rather than updating, that is controlled and difficult.

Here we show that updating can be fast or slow, depending on what is updated. Specifically, resolving the above discrepancy relies on differentiating between updating items and item-context associations. Typical updating tasks require participants to keep track of the most recent item that corresponds to each of several contexts, being locations on the screen (Ecker et al., 2010; Kessler & Meiran, 2006, 2008; Oberauer 2002), serial positions (Kessler & Oberauer, 2014, 2015; Morris & Jones 1990), or semantic categories (Miyake et al., 2000). These tasks involve the simultaneous maintenance of several items that differ in their associated context. In these tasks, the context in WM is preserved throughout a sequence of trials, but the content that is bound to it is updated from one trial to another. Therefore, correct performance is dependent on forming, maintaining, and retrieving these item-to-context associations. Notably, these processes are different from the encoding of new information that often requires also the formation of a new context representation, such as in the case of binding new items to new spatial positions in a visual WM task, or new words to new syntactic structures during language comprehension.

For example, in the n-back task, participants must compare the item presented on the screen to the one that appeared n trials before. Even in its easiest variant, the 1-back task, in which each item should be compared to the immediately preceding one, two items should be held in WM during the comparison process: the present and the previous one. Confusing between them is detrimental to task performance. Hence, each item should be associated with its role or context, being the present or previous item. It follows that this and other updating tasks require updating item-context binding rather than items per se. While updating these bindings is costly, we suggest that merely updating items without the need to bind them into a context is seamless and even obligatory, a by-product of their attention-consuming processing.

To test this idea, we designed a minimal updating task. Experiment 1 required maintaining either 1 (set-size = 1; SS1) or two (set-size = 2; SS2) items in WM (see Figure 1). In each trial, participants performed a choice reaction-time (RT) task, in which they had to decide, using a keypress, whether the presented item is X or O. Unlike many other updating tasks that require a same/different decision, responding correctly in our task did not depend on remembering any of the items that appeared in previous trials, but only on the currently presented letter. In addition to the choice task, in each trial, the participants were prompted whether to update the presented item into WM or not. After a few trials, they had to recall the item held in their WM. While minimal in its processing requirements, this task shares all the features of more complex updating tasks: holding items in memory over several trials, selectively updating them upon demand, frequently updating the information throughout a sequence of trials, and filtering out distractors when updating is not required. To anticipate the results, updating was slower than no-updating in SS2, reflecting the need to associate each item to its context and update these associations in update trials. However, updating was faster in SS1, in which no item-binding associations needed to be updated.

**Figure 1:**
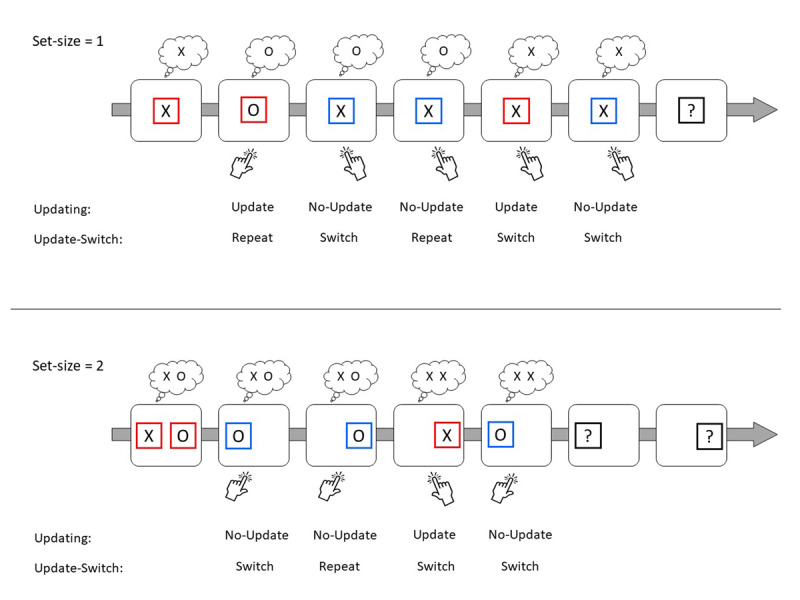
Experiment 1: Trial sequences in SS1 (top) and SS2 (bottom). In each trial, participants are presented with one letter and need to respond to it using their right/left index finger. In addition, participants need to remember the letter that appeared in the most recent red frame, for a probed recall at the end of the sequence. Red/blue frames indicate the update/not-update conditions, respectively. Bubbles represent the content of WM in each trial. Each stimulus appeared on the screen until a response was indicated. The inter-trial interval was 500 ms.

## Experiment 1

### Method

#### Participants

Thirty-four students from Ben-Gurion University of the Negev participated in the experiment in return for course credit. Nine participants were excluded from the analysis due to low accuracy (<80%) in either the choice RT trials or the recall phase. 25 participants (22 women, age: *M =* 23.280, *SD* = 2.441) were therefore included in the analysis. The experiment was approved by the Ethics Committee of the Department of Psychology, Ben-Gurion University of the Negev.

#### Procedure

Stimuli presentation was programmed in OpenSesame (Mathôt, Schreij & Theeuwes, 2012). The study was run using JATOS (Lange, Kühn & Filevich, 2015). Participants performed the experiment online using their personal computers through their internet browsers.

The experiment was composed of two parts, corresponding to SS1 and SS2, respectively. 25 sequences of trials were administered in SS1 and 50 sequences in SS2. Each part was preceded by two practice sequences.

Participants performed a choice RT task (Figure 1). In each trial of SS1, the letter X or O appeared on the screen, and the participants needed to respond to it with the keys *p* or *q*, respectively. The stimulus appeared on the screen until the response was indicated. Each letter appeared within a red or a blue frame. Throughout each sequence, participants were required to remember the letter that appeared in the most recent red frame, and to report it at the end of the sequence. Accordingly, a red frame required updating WM with the letter that appeared inside it (“update” condition), while a blue frame did not (“no-update” condition). The first trial in each sequence was always an update trial. The updating condition in each of the subsequent trials was chosen at random with equal probabilities for update and no-update. The inter-trial interval was 500 ms. Each trial had a 10% probability of being the last in the sequence. Accordingly, the number of trials in each sequence followed a geometric distribution, so that the participants could not expect the end of the sequence. A recall probe appeared at the end of each sequence, in the form of a question mark within a black frame. The participants needed to indicate the letter they remember, namely the last letter that appeared within a red frame, using the *x* and *o* keys.

SS2 was similar but included two frames, one presented on the left side of the screen and the other on the right. In the first trial, both frames were presented in red, with the letter X or O within each of them. Then, in each trial, one of the frames appeared on the screen in either red or blue, with a letter inside. Participants were required to respond to the letter (as done in SS1), and remember the last letter that appeared within a red frame on each of the sides. Accordingly, two letters needed to be memorized throughout the sequence. After a random number of trials, the participants were prompted to recall the most recent red letter that appeared in each side in random order using the X and O keys.

#### Design and Analysis

The main analysis focused on RT in the choice RT task as a function of Set-Size (1 vs. 2) and Updating (update vs. no-update). Parallel analyses of accuracy in the choice task are reported to rule out the possibility of a speed-accuracy tradeoff. One difference between the set-sizes is that in SS2 the present item could appear in the same frame as the previous item, or in a different frame. The latter situation requires switching the focus of attention from one item to another (Garavan, 1998; Oberauer, 2002), a process that might interact with Updating. To remove this confound, the analysis of SS2 only involved item-repetition trials, namely trials in which the letter appeared in the same frame as the previous item (but did not necessarily involve the same letter). We also examined the effect of Update-Switch, defined as whether or not the updating condition in Trial N differed from Trial N-1. Switch trials are update trials following no-update trials or vice versa. Repeat trials are update following an update, and no-update following a no-update. This variable is interesting based on the finding that switching between update and no-update trials results in a substantial behavioral cost, attributed to switching the state of the gate to WM (Kessler & Oberauer, 2014; Rac-Lubashevsky & Kessler, 2016a, b). Additional analyses examined performance as a function of lag-2 switch vs. repetition (Experiment 3) and in the recall phase (Experiments 2 and 3). The analysis only included trials from sequences in which the final recall step was correct. Error and post-error trials were removed from the RT analysis. RTs that deviated more than 2sd from the mean of their condition within each subject were dismissed as outliers. All analyses were carried out in R (R Core Team, 2017) using the RStudio IDE (RStudio team, 2020; version 4.1.2) using “afex” (Singmann et al., 2021; version 1.0-1), “emmeans” (Lenth, 2022; version 1.7.2), and “tidyverse” (Wickham et al., 2019; version 1.3.1) packages.

### Results

#### Choice trials RT

**Figure 2:**
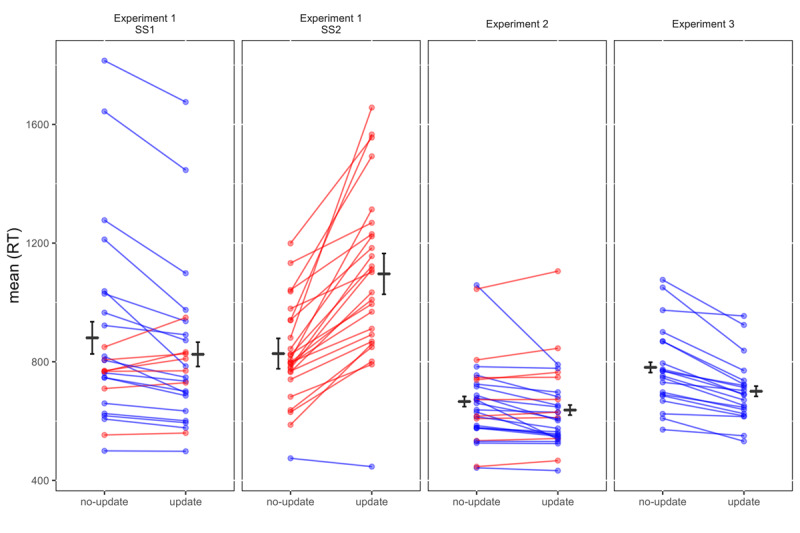
RT by Updating for Experiments 1, 2, and 3. Dots connected by horizontal lines represent individual participants. The line color represents the slower condition for each participant. Red lines represent participants for which updating was slower than not-updating (namely, with an updating cost), whereas participants showing faster updating than not-updating (an updating benefit) are represented by blue lines. Horizontal black lines represent grand averages. Error bars represent within-subject confidence intervals. SS = Set-Size.

An ANOVA was conducted with Set-Size (1,2), Updating (update, no-update), and Update-Switch (switch, repeat) as within-subject variables. Significant main effects were observed for Set-Size, *F(1,24) = 10.64, p < .001, η_p_^2^ = .31*, Updating, *F(1,24) = 37.33, p < .001, η_p_^2^ = .61*, and Update-Switch, *F(1,24) = 51.19, p < .001, η_p_^2^ = .68*. The two-way interaction between Updating and Update-Switch was significant, *F(1,24) = 9.51, p = .005, η_p_^2^ = .28*, reflecting a larger switching effect in update trials (295 ms) than in no-update trials (201 ms). The interaction between Set-Size and Update-Switch was also significant, *F(1,24) = 16.63, p < .001, η_p_^2^ = .41*, reflecting a larger switch cost in SS2 compared to SS1, 298 vs. 197 ms, respectively. The main finding of interest was a significant two-way interaction between Updating and Set-Size, *F(1,24) = 64.09, p < .001, η_p_^2^ = .73* (see Figure 2). In SS2, update trials were 279 ms *slower* than no-update trials, *F(1,24) = 61.37, p < .001*. In SS1, by contrast, update trials were 49 ms *faster* than no-update trials, *F(1,24) = 8.37, p = .008*. The three-way interaction was non-significant, *F(1,24) = 2.61, p = .119, η_p_^2^ = .10*.

### Choice trials accuracy

The main effect of Updating was significant, *F(1,24) = 20.06, p < .001, η_p_^2^ = .46*, reflecting a larger error proportion in no-update trials (4.5%) than in update trials (2.6%). The main effect of Set-Size was significant, *F(1,24) = 8.34, p = .008, η_p_^2^ = .26*, reflecting a larger error proportion in SS2 (4.6%) than in SS1 (2.6%). Also, a significant main effect for Update-Switch, *F(1,24) = 5.74, p = .025, η_p_^2^ = .19*, reflected a larger error proportion in repetition than in switch trials, 4.2% vs. 3%, respectively. Finally, the two-way interaction between Updating and Update-Switch was significant, *F(1,24) = 8.75, p = .007, η_p_^2^ = .27*. Specifically, the simple main effect of Updating was significant in repetition trials, *F(1,24) = 19.02, p < .001*, but not in switch trials, *F(1,24) = 3.56, p = .071*.

## Experiment 2

Our results show that updating is slower than not-updating when item-position associations are required, namely in SS2. However, it is quicker than not-updating when a single item needs to be maintained. Whereas the longer updating latencies in SS2 are consistent with previous findings in the WM updating literature, the finding in SS1 is novel and surprising. The goal of Experiment 2 was to replicate this finding.

### Method

#### Participants

Thirty students from Ben-Gurion University of the Negev participated in the experiment in return for course credit. Two participants were excluded from the analysis due to low accuracy (<80%) in either the choice RT trials or the recall phase. 28 participants (16 women, age: *M =* 23.250, *SD* = 1.379) were therefore included in the analysis. The experiment was approved by the Ethics Committee of the Department of Psychology, Ben-Gurion University of the Negev.

#### Procedure

The procedure was identical to that of Experiment 1, except for the following changes. First, this experiment only examined the SS1 condition, since this is that condition in which the novel finding was observed in Experiment 1. Second, the number of trial sequences in SS1 was increased to 55. Third, the same response keys were now used for both the choice task and the recall phase, namely p and q for X and O, respectively. Accordingly, the participants did not need to move their fingers between the choice and recall trials, enabling to measure RT at recall. Unlike Experiment 1, this experiment was conducted in the lab.

### Results

#### Choice trials RT

The main effect of Updating was significant, *F(1,27) = 5.78, p = .023, η_p_^2^ = .18*, reflecting faster RTs in update than in no-update trials (638 vs. 666 ms, respectively). This finding replicates Experiment 1. Also, the main effect of Update-Switch was significant, *F(1,27) = 47.85, p < .001, η_p_^2^ = .64*, reflecting a switch cost of 101 ms. The 2-way interaction was non-significant, F(1,27) < 1.

#### Choice trials accuracy

The main effect of Updating was significant, *F(1,27) = 12.96, p = .001, η_p_^2^ = .32*, as well as the two-way interaction, *F(1,27) = 5.36, p = .028, η_p_^2^ = .17*. Specifically, update trials were more accurate than no-update in repetition trials (3.8% vs. 6.4%, respectively), *F(1,27) = 14.42, p < .001*, but the two did not significantly differ in switch trials (4.3% vs. 5.0%, respectively), *F(1,27) = 1.73, p = .20*.

#### Recall performance

We examined RT in the recall trials to see whether the duration of recall was sensitive to the number of trials that took place since the presentation of the last red frame. Such a finding could imply that participants did not update their WM throughout the trial sequence but rather retrospectively tried to recall the letter in the last red frame when prompted to do so. Such a reactive control strategy (Braver, 2012) is already implausible since it predicts no RT difference between update and no-update trials during the choice trials. Still, we aimed to test its prediction that RT will be longer the further away was the most recent red frame. To test this, we examined RT for probes that appeared following 1, 2, or ≥3 no-update trials in a row.

Probes that immediately followed an update trial were excluded from the analysis, because responding to the probe in these cases involves a stimulus- and response-repetition. RTs for correct probes following 1, 2, and ≥3 no-update trials in a row were 785, 831, and 805 ms. Apart from being non-monotonic, this trend was clearly non-significant, *F(2,54) = 1.28, p = .287, η_p_^2^ = .05*. The effect on recall accuracy was also non-significant, *F(2,54) = .13, p = .881*. These results support the idea that updating took place in the choice trials and not retroactively.

## Experiment 3

Why is updating faster than not updating? One possibility is that attending to an item during response selection leads to its updating into WM in an obligatory manner, whether updating is required or not. Removal (Lewis-Peacock, Kessler & Oberauer, 2018) then applies to the items that did not require updating, namely those in the blue frames, to prevent interference with the goal-relevant content of WM, namely the letter in the most recent red frame. We capitalized on the lag-2 repetition effect (Kessler, 2018; Mayr & Keele, 2000) to examine this possibility. If removal takes place, then responding to an item that appeared two trials before across a different intervening item (e.g., X preceded by O that was preceded by X; n-2 repetition) is expected to be slower than responding to an item that differed from the one that appeared two trials ago (e.g., X preceded by O that was preceded by Z; n-2 switch). This is because moving from X to O entailed suppressing X as part of its removal from WM, which impairs the response to X in the following trial. Accordingly, a removal account of our results predicts n-2 repetition cost in no-update trials.

### Method

#### Participants

Thirty-two students from Ben-Gurion University of the Negev participated in the experiment in return for course credit. Twelve participants were excluded from the analysis due to low accuracy (<80%) in either the choice RT trials or the recall phase. 20 participants (18 women, age: *M =* 23.60 *SD* = 1.27) were therefore included in the analysis. The experiment was approved by the Ethics Committee of the Department of Psychology, Ben-Gurion University of the Negev.

#### Procedure

The procedure of Experiment 2 was used with the following changes. In each trial, the letter X, O or Z appeared on the screen, and the participants needed to respond with the keys *j, k* and *l*, respectively, using their index, middle, and ring fingers. In order to ensure we get enough sequences for the analysis, each trial had an 80% chance of having the same colored frame (namely, Updating condition) as the previous trial. In addition, each sequence had a minimum of 3 trials. The experiment was conducted online as in Experiment 1.

### Results

#### Choice trials RT

An ANOVA was conducted with Updating and Update-Switch as within-subject variables. The main effect of Updating was significant, *F(1,19) = 25.26, p < .001, η_p_^2^ = .57*, again demonstrating faster RTs in update than no-update trials, 733 vs. 835 ms, respectively. The main effect of Update-Switch was also significant, *F(1,19) = 25.99, p < .001, η_p_^2^ = .58*, reflecting longer RTs in switch than in repetition trials, 862 vs. 705 ms, respectively. The two-way interaction was non-significant, *F(1,19) = 3.56, p = .074, η_p_^2^ = .16*.

#### Choice trials accuracy

None of the main effects was significant, *F(1,19) = 2.18, p = .157, η_p_^2^ = .10* for Updating (3.1% vs 3.6% errors in the update and no-update conditions, respectively), and *F(1,19) = .42, p = .523, η_p_^2^ = .02* for Update-Switch. The two-way interaction was also non-significant, *F(1,19) = 3.60, p = .073, η_p_^2^ = .16*.

#### Lag-2 repetition RT

**Figure 3:**
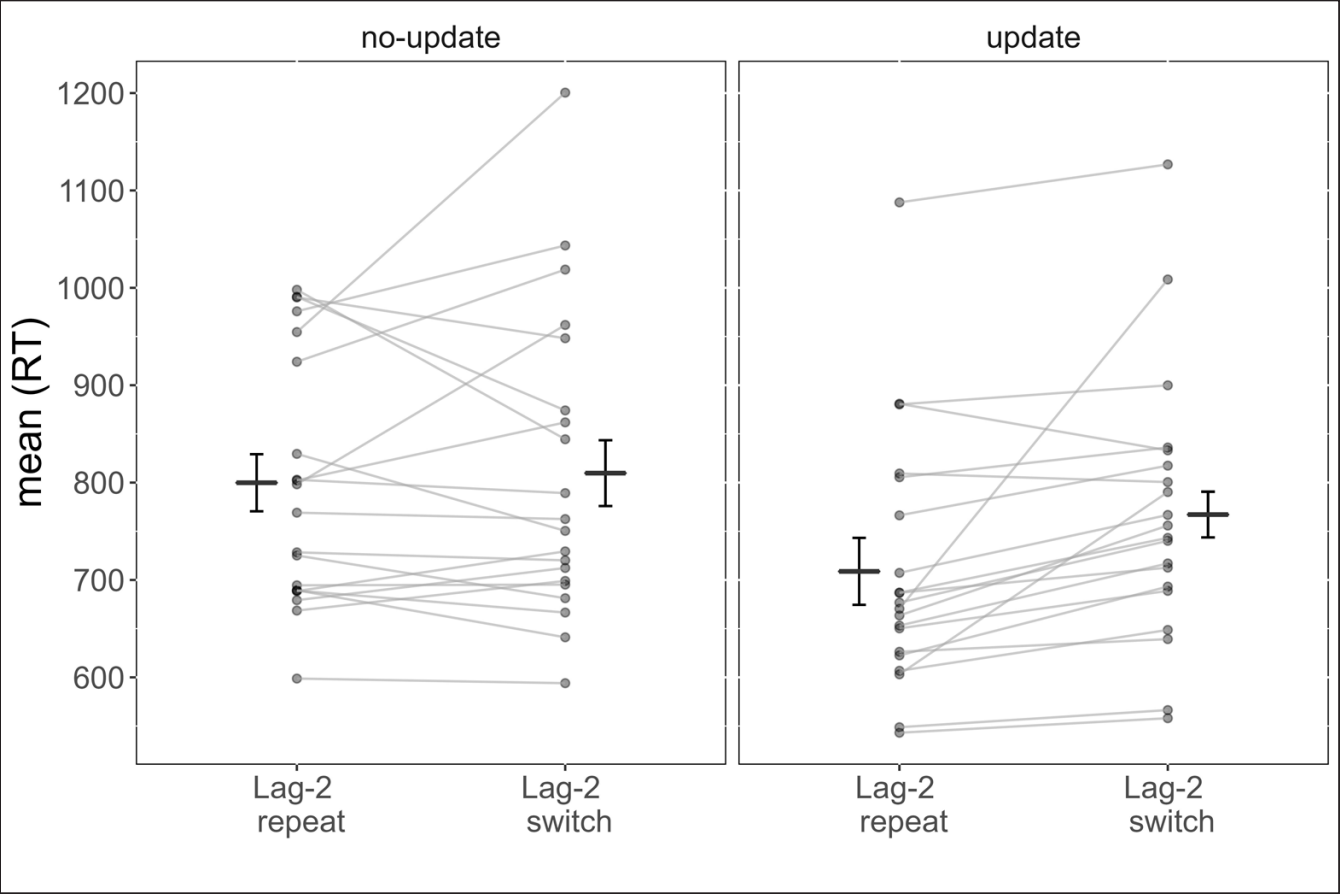
Experiment 3: Lag-2 repetition benefit was found in update trials, whereas no effect was observed in no-update trials. Horizontal black lines represent grand averages across participants. Error bars represent within-subject confidence intervals.

Only trials that were preceded by at least two trials of the same updating condition (either update or no-update) entered the analysis. We compared lag-2 repetition trials (e.g., XOX) to switch trials (e.g., ZOX). An ANOVA was conducted with Updating and Lag-2 as within-subject variables. The main effect of Updating was significant, *F(1,19) = 16.40, p < .001, η_p_^2^ = .46*, reflecting faster RTs in update than no-update trials, 738 vs. 805 ms, respectively. The effect of Lag-2 was non-significant, *F(1,19) = 3.79, p = .067, η_p_^2^ = .17*. However, the 2-way interaction was significant, *F(1,19) = 9.91, p = .005, η_p_^2^ = .34* (see Figure 3). Specifically, the simple main effect of Lag-2 in update trials was significant, *F(1,19) = 10.59, p = .004*, reflecting faster RTs in lag-2 repetition trials than in switch trials, 709 vs. 767 ms, respectively. The simple main effect of Lag-2 in no-update trials was non-significant, *F(1,19) = .24, p = .632*.

#### Lag-2 repetition accuracy

Only the main effect of Lag-2 was significant, *F(1,19) = 5.63, p = .028, η_p_^2^ = .23*, reflecting more errors in repetition trials (4.5%) than in switch trials (2.7%). However, this was not qualified by a 2-way interaction, *F(1,19) = 1.08, p = .312, η_p_^2^ = .05*. The main effect of Updating was also non-significant, *F(1,19) = .79, p = .384, η_p_^2^ = .04*.

#### Recall performance

Six participants were removed from the RT analysis and 2 from the error analysis due to empty cells. RTs were 947, 922 and 851 ms for probes following 1, 2, or ≥3 no-update trials in a row, respectively, *F(2,26) = 1.36, p = .274, η_p_^2^ = .10*. Error rates 14.7, 5.9 and 15.3%, respectively, *F(2,34) = 1.26, p = .297, η_p_^2^ = .07*. As in Experiment 2, no evidence was found to support a retroactive control strategy.

### Discussion

The basic finding of quicker update RTs was again replicated. Lag-2 repetition facilitated performance in update trials. This observation is consistent with the idea that removal does not take place in these trials, and hence an item that appeared two trials before can prime the present item and facilitate its processing. We hypothesized that this effect will be reversed in no-update trials. However, contrary to our prediction, such a reversal was not observed. Nevertheless, the abolished repetition gain in this condition is consistent with the notion of suppressing not-updated information. A similar result was reported by Ecker et al. (2014), who found that the repetition benefit in a memory updating paradigm was reduced but not eliminated, following a removal cue. Future work should further examine the removal account of no-update slowing.

## General Discussion

Using a “minimal” updating task, the present study showed, for the first time, that WM updating is fast and obligatory when only one item is held in mind. This surprising result is at odds with the common finding in the literature, showing that updating is rather slow and controlled. For example, Kessler and Meiran (2008) presented participants with strings of letters and asked them to update their WM with the most recent one, that had to be recalled at the end of the sequence. The number of repeated vs. updated letters in each trial was manipulated. The results showed that the latency of WM updating increases with both set-size and the number of updated items. Also, updating the entire set-size was quicker than updating part of the set. However, trials in which updating was not required at all was even quicker than a whole-set updating. This RT cost was later attributed to the serial updating of item-position associations (Kessler & Oberauer, 2014, 2015). Additional work using the reference-back task (e.g., Rac-Lubashevsky & Kessler, 2016) also showed consistently that updating is slower and more error prone than not-updating.

The finding of slow and effortful updating is consistent with previous theorizing addressed the apparent conflict between the role of WM in shielding the maintained information against interference and its effective updating with goal-relevant information upon need (Frank, Loughry & O’Reilly, 2001; Hazy, Frank & O’Reilly, 2007; O’Reilly, Braver & Cohen, 1999). Ample work suggests that these seemingly opposing computational goals are coordinated by a gating mechanism that regulates the flow of information into WM (Braver & Cohen, 2000; Chatham & Badre, 2015; McNab & Klingberg, 2008). The gate is closed by default to protect the maintained information against interference and is transiently opened to update WM with new, goal-relevant information. Control over the gate state is associated with activation in dopaminergic brainstem nuclei (D’Ardenne et al., 2012) and in the basal-ganglia (Nir-Cohen et al., 2020). Since the default gate-state is closed, updating requires a cascade of processes that include opening the gate and encoding the new information, possibly while forming new item-context associations and removing old ones. Carrying these processes out is time-costly and non-obligatory. In contrast to the above, we show that under simple conditions that involve the concurrent processing and maintenance of one item only, updating is rather quick and obligatory.

Our results are consistent with the view that attending to an item, for example as required for response-selection, results in its obligatory encoding into WM. In other words, updating of a single item is carried out as part of drawing attention to it. Hence, the updating gain showed in SS = 1 is not due the facilitation of the updating condition, but rather due the slow-down of the no-update condition. Specifically, items that were obligatorily updated during response selection, but do not need to be further maintained (i.e., appear in the no-update condition), need to be removed. This is reflected by a diminished lag-2 repetition benefit in the no-update condition. Since removal takes time (Oberauer, 2001; Ecker et al., 2014), the duration of no-updating is slowed.

The finding of an update-switch cost in SS1 calls for a reinterpretation of this effect. Previous studies have shown that switching between no-update and update trials, or vice versa, results in a substantial RT cost, ascribed to opening or closing the gate, respectively (Kessler & Oberauer, 2014; Rac-Lubashevsky & Kessler, 2016b; Rac-Lubashevsky, Slagter & Kessler, 2017). Our findings of obligatory updating in SS1 imply that the gate remained open throughout performance, both in updating and no-updating trials, and hence the need for removal in no-update trials. Accordingly, the finding of an update-switch cost in SS1 thus cannot be attributed to gating, and presumably reflects switching between two tasks, namely updating and not-updating. Previous findings using the reference-back task indicated that update-switching is not merely task-switching, as demonstrated by asymmetrical brain activity when switching in the two directions (Nir-Cohen et al. 2020; Rac-Lubashevsky & Kessler, 2018). Also, update-switching was observed even in tasks where the difference between update and no-update trials was not made explicit to the participant (Kessler & Oberauer, 2014). However, based on the findings in SS1, we contend that the RT cost of update-switch in earlier studies may partially reflect task switching.

To conclude, we identified a condition in which updating is the default mode of WM. When holding only one item in mind, updating is a by-product of attention, which in our case is manifested in the response selection as part of the choice RT task. Notably, recent evidence had shown that when given the opportunity to choose the number of items held in WM, people tend to rely on one item only (Draschkow, Kallmayer & Nobre, 2021). Accordingly, the situation of our SS1 condition is very common during natural “real world” behavior, implying that most of the time, updating our WM is fast and effortless.

## Data accessibility statements

This study was supported by an Israel Science Foundation grant #1088/21 awarded to Y.K. The raw data and analysis codes for this study are available at https://osf.io/vt9xk/.
